# Health-care associated infection in Africa

**DOI:** 10.1186/1753-6561-5-S6-O14

**Published:** 2011-06-29

**Authors:** S Bagheri Nejad, B Allegranzi, SB Syed, B Ellis, D Pittet

**Affiliations:** 1 World Health Organization, Geneva, Switzerland; 2 University of Geneva Hospitals and Faculty of Medicine, Geneva, Switzerland

## Introduction / objectives

To assess the epidemiology of endemic health care-associated infection (HAI) in Africa.

## Methods

We searched biomedical databases to identify studies published from 1995 to 2009 on the epidemiology of HAI in African countries. No language restriction was applied. Available abstract books of leading international infection control conferences were also searched from 2004 to 2009.

## Results

Nineteen articles met the eligibility criteria for inclusion in the review; only two met high-quality criteria. Four relevant abstracts were retrieved from the international conference literature. Hospital-wide HAI prevalence varied between 2.5 and 14.8 per 100 patients; in surgical wards, the cumulative incidence ranged from 5.7 to 45.8 per 100 patients. Among specific types of infection, the largest number of studies focused on surgical site infection with a cumulative incidence ranging from 2.5 to 30.9 per 100 operated patients. Data on causative pathogens were available from a few studies only and highlighted the importance of Gram-negative rods, particularly in surgical site infection and ventilator-associated pneumonia.

## Conclusion

Limited information is available on the endemic burden of HAI in Africa, but our review reveals that its frequency is several-fold higher than in developed countries. There is an urgent need to identify and implement feasible and sustainable approaches to strengthen HAI surveillance and control in Africa, including preventive strategies.

## Disclosure of interest

None declared.

